# A novel polymeric fibrous microstructured biodegradable small-caliber tubular scaffold for cardiovascular tissue engineering

**DOI:** 10.1007/s10856-021-06490-1

**Published:** 2021-03-01

**Authors:** Andreas Dimopoulos, Dionysios N. Markatos, Athina Mitropoulou, Ioannis Panagiotopoulos, Efstratios Koletsis, Dimosthenis Mavrilas

**Affiliations:** 1 Department of Mechanical Engineering and Aeronautics, Laboratory of Biomechanics and Biomedical Engineering, University of Patras, Patras, GR Greece; 2 University Hospital, Cardiothoracic Surgery Clinic, University of Patras, Patras, GR Greece

## Abstract

Increasing morbidity of cardiovascular diseases in modern society has made it crucial to develop artificial small-caliber cardiovascular grafts for surgical intervention of diseased natural arteries, as alternatives to the gold standard autologous implants. Synthetic small-caliber grafts are still not in use due to increased risk of restenosis, lack of lumen re-endothelialization and mechanical mismatch, leading sometimes either to graft failure or to unsuccessful remodeling and pathology of the distal parts of the anastomosed healthy vascular tissues. In this work, we aimed to synthesize small-caliber polymeric (polycaprolactone) tissue-engineered vascular scaffolds that mimic the structure and biomechanics of natural vessels. Electrospinning was implemented to prepare microstructured polymeric membranes with controlled axis-parallel fiber alignment. Consequently, we designed small-caliber multilayer anisotropic biodegradable nanofibrous tubular scaffolds, giving attention to their radial compliance. Polycaprolactone scaffold morphology and mechanical properties were assessed, quantified, and compared with those of native vessels and commercial synthetic grafts. Results showed a highly hydrophobic scaffold material with a three-layered tubular morphology, 4-mm internal diameter/0.25 ± 0.09-mm thickness, consisting of predominantly axially aligned thin (1.156 ± 0.447 μm), homogeneous and continuous microfibers, with adequate (17.702 ± 5.369 μm) pore size, potentially able to promote cell infiltration in vivo. In vitro accelerated degradation showed a 5% mass loss within 17–25 weeks. Mechanical anisotropy was attained as a result, almost one order of magnitude difference of the elastic modulus (18 ± 3 MPa axially/1 ± 0.3 MPa circumferentially), like that of natural arterial walls. Furthermore, a desirable radial compliance (5.04 ± 0.82%, within the physiological pressure range) as well as cyclic stability of the tubular scaffold was achieved. Finally, cytotoxicity evaluation of the polymeric scaffolds revealed that the materials were nontoxic and did not release substances harmful to living cells (over 80% cell viability achieved).

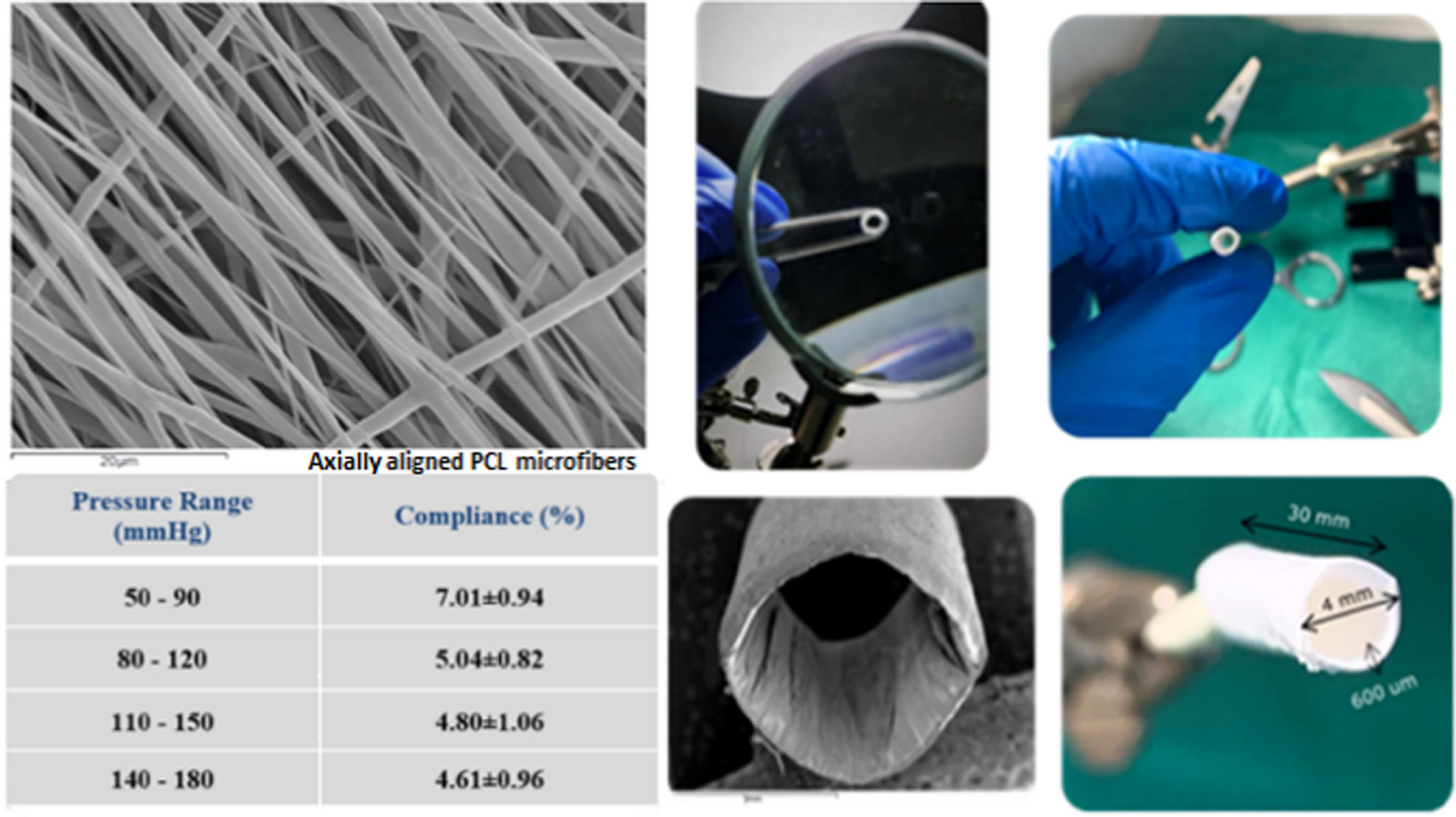

## Introduction

Cardiovascular diseases (CVDs) are the main cause of death in industrialized countries and among the top three worldwide. In Europe, 3.9 million deaths annually (1.8 million in the EU alone) are owed to CVDs, while the total costs are calculated for the EU in 210 billion euros annually (2017 statistics) [1]. The increasing need for surgical therapies of CVDs in the modern society has made it crucial, among others, to develop blood vessel substitutes especially for diseased small-caliber ones (<6 mm).

Although autologous vessels remain the gold standard for small-caliber grafts, inability to use autografts due to vessel pathologies or previous surgical interventions, can limit their clinical application. On the other hand, limited availability of homografts due to unavailable tissue donors makes sometimes implantation of living blood vessels a difficult task [2, 3].

Prosthetic grafts, such as polytetrafluoroethylene (ePTFE, a.k.a. Gore-Tex) and poly(ethylene terephthalate) (PET, a.k.a. Dacron), have been clinically used alternatively as replacements of large diameter arteries. However, restenosis, infection, thrombosis, lack of re-endothelialization, compliance and other biomechanical mismatch to the anastomosed native blood vessels are common problems within these synthetic grafts [4–8]. Among other biomechanical properties radial compliance, suture retention strength and cyclic stability are critical for a prolonged graft patency. Mismatch in radial compliance (a measure of the graft diameter change under a pressure range) between the graft and the end to end anastomosed native vessel may cause, among other problems, local increase in shear stress disturbances that may induce intimal hyperplasia and ultimately graft failure [9].

Over the last few decades, vascular tissue engineering (VTE) has emerged as an alternative approach to overcome the limitations of the afore-mentioned vascular grafts [10]. An ideal tissue-engineered vascular scaffold should completely integrate with the surrounding vessels and function as a 3D framework for host infiltration of undifferentiated mesenchymal stem cells as well as differentiated cells (endothelial, smooth muscle cells, and fibroblasts), in order to regenerate the vascular extracellular matrix (ECM) in vivo. To provide an adequate 3D structure and enable full integration into the site of the damaged vessels, a biomimetic scaffold should fulfill the following requirements: (a) mimic the physicochemical and biochemical properties of natural blood vessels ECM, (b) be biocompatible, (c) demonstrate enough porosity and porous size, to allow host cell infiltration and endovascular lumen formation when needed, avoiding, however, hemorrhage, (d) present appropriate mechanical properties (e.g., matching radial compliance to the native vessels, high suture retention strength, and cyclic stability), and (e) degrade safely in synchronization with the newly growing tissue [11, 12].

Acellular xenogeneic ECM scaffolds have been utilized for VTE applications. Different decellularization techniques, such as glutaraldehyde or alternative agent tissue fixation, have been used since the 60s for biological heart valves. However, tissue calcification, leading to stiffening and severe dysfunction, is a common problem following tissue fixation. The lack of any regeneration ability makes the fixed tissues unsuitable candidates for VTE. Non-fixed xenogeneic tissues, although used in different cardiovascular applications, are, currently, not used for the construction of small-caliber blood vessels due to a combination of factors needed to overcome all failure mechanisms [13].

Synthetic biodegradable polymeric scaffolds are alternatives for scaffold design. The freedom to utilize polymer synthesis and 2D or 3D construction makes them challenging to design a scaffold with predetermined geometry and internal structure. Hydrophobic and hydrophilic polymers can be used in different combinations to successfully achieve final geometrical, structural, biomechanical, and biocompatibility characteristics of a small-caliber VTE scaffold.

Many research groups have focused on the design of VTE grafts that mimic the structure of natural blood vessels by using different scaffold fabrication techniques such as molecular self-assembly, solvent-casting/particulate-leaching technique, thermally induced phase separation, and electrospinning [14]. In recent years, the interest on the electrospun scaffolds for the development of VTE has increased tremendously [15]. Although studies on this field are currently in the early stages, recent reports have highlighted the potential use of electrospun scaffolds due to their tunable mechanical and biological properties [16–21]. Among others, electrospinning has a great potential to mimic vascular tissue ECM architecture, composed of three-layer organized 50–500-nm diameter fibrous proteins. Electrospinning offers the ability to fine-tune mechanical properties during the fabrication process, while also controlling the necessary biocompatibility and structure of the tissue-engineered grafts. The ability of electrospinning technique to combine the advantages of synthetic and natural materials makes it attractive for tissue engineering applications where a high mechanical durability, in terms of cyclic stability and radial compliance, is required. Furthermore, incorporation of natural polymers, with abundance of cell binding sites, can promote the formation of a continuous monolayer of EC in the lumen and proliferation of other cell types in the matrix of the graft’s wall. The electrospinning technique also offers precise control over the composition, dimensions, and alignment of fibers that have impact on the porosity, pore size distribution, and the architecture of scaffolds. This method allows for engineering of a wide range of tunable structural and mechanical properties as required for specific applications.

The mechanical properties of electrospun scaffolds are controlled by changing various microstructural parameters such as fiber diameter, porosity, and alignment. In addition to uniaxial tensile properties, the burst strength and compliance are also important mechanical properties for vascular grafts. However, the latter properties are rarely measured and reported. Even though electrospinning process has numerous advantages over other methods, challenges still remain that need to be overcome prior to the clinical use of electrospinning in tissue-engineered vascular grafts (TEVG). The major issues associated for the application of electrospun scaffolds, particularly for TEVG include: lack of adequate cell penetration in thick scaffolds due to small pore sizes; poor surface properties that may have negative impact on the cell viability, proliferation, and growth; shortage of favorable cells; poor control over mechanical properties and degradation; biological response of the tissues/cells toward TEVGs [10, 22]. While robust physical and mechanical properties, including burst strength, water permeability, and suture strength, are required for grafting, high compliance is critical, because the compliance mismatch between a vascular graft and neighboring arteries at the site of anastomosis is a major cause of graft failure [10, 15]. Thus, selecting an appropriate polymer is one of the most essential parameters of vascular graft tissue engineering to overcome the above limitations.

TEVG from electrospun PET-PU [23] or polylactine/gelatin [24] have been proposed as small-diameter vessel substitutes with satisfying biological and mechanical characteristics. Among other biocompatible biodegraded polymers, PCL has been proven an ideal component of long-term implant materials to produce blood vessel scaffolds [10, 15] due to its slow degradation rate (>2.5 years) in human body. PCL chemically degrades due to hydrolytic cleavage of the back-bone ester bonds and thereby converting long polymer chains into shorter water-soluble fragments. Electrospun membranes made of polycaprolactone (PCL) and its composites are widely used in biomedical applications, especially as scaffolds in tissue-engineering applications and have been shown to possess excellent biomechanical features and structural similarity with the ECM of blood vessels [9, 10, 15].

The current work is a pilot study toward the design of electrospun anisotropic biodegradable nanofibrous small-caliber polymeric VTE scaffolds that mimic the structure and biomechanics of natural blood vessels, giving emphasis to their radial compliance. We used PCL, a hydrophobic highly biocompatible and biodegradable (FDA-approved) aliphatic polyester as the high-strength material of a multilaminate final design that can support the creation of a polymer-cell complex in vitro with subsequent implantation in vivo [25].

## Materials and methods

### Preparation of the polymer solution

Twenty percent w/v PCL pellets (Mn 80,000, Sigma-Aldrich, 440744) were dissolved in glacial acetic acid (≥99.8%, Honeywell Fluka). The solution was transferred in 20-ml cylindrical plastic vials and stirred with a laboratory roller mixer for 24 h under 40 °C in order to make a transparent solution. The solution was then left to cool at room temperature and used within 2 days.

### Scaffold fabrication by electrospinning

An in-house electrospinning setup was used in this study. A 10-ml syringe with a plastic tube extension and a 20G (inner diameter 0.6 mm) stainless steel blunt needle at the end was filled with the PCL solution and driven by a syringe pump (New Era Pump Systems Inc. NE-1000), at a feed rate of 2 ml/h. A high DC voltage of 20 kV (Spellman SL300 DC power supply unit) was applied between the metallic needle (+) and a rotating aluminum cylindrical drum collector surface (earth), 5-cm width and 10-cm diameter, at a distance of 22 cm. A custom-designed and constructed Arduino-driven (Arduino Mega 2560 Rev3 with motor shield Rev3) was used to accurately control the angular velocity of the drum [17]. The average temperature and humidity recorded during the experimental process was 20–25 °C and 50–60%, respectively. After preliminary experiments, to achieve better fiber quality and peripheral alignment on the cylindrical surface, a rotational drum speed of 1280 rpm was selected for the electrospinning process. The collector surface was covered with aluminum foil to collect and easily detach the electrospun membranous scaffolds, used to form the small-caliber tubular scaffolds in a later stage. Electrospinning was performed for 2 h.

### Scaffold morphology assessment

The morphology of the fibrous scaffold was observed by Scanning Electron Microscopy (SEM) (JEOL 6300). Rectangular-shaped specimens were gold-sputtered prior to SEM observation. Based on the SEM images, fiber alignment was assessed and the fiber diameter, as well as the pore size, was measured using an image processing software (Image J, version 1.51n, National Institutes of Health, USA).

### Contact angle measurements

To determine wettability of the PCL scaffolds and possible alterations associated with the electrospinning procedure and fiber morphology and orientation, contact angle measurements (sessile drop method) were performed at room temperature at a contact angle meter (CAM 101, KSV instruments Ltd.) using phosphate buffer saline (PBS, pH 7.4) as the test liquid. Scaffolds were freeze-dried prior to the wettability measurements and six (6) specimens 10 × 10 mm were tested.

### In vitro degradation measurements

An accelerated degradation test in PBS was used to assess the degradation properties of the scaffold material. To achieve that, rectangular specimens 10 × 10 mm from the scaffolds were freeze-dried and their initial weight (WI) was recorded. After that, they were placed in 20-mL plastic vials, each containing 10 mL of 0.1 M, pH 7.4 PBS and immersed into a stirred water bath at 37 °C for a total period of 28 weeks. Every 2 weeks specimens (*n* = 3) were taken out, washed three times with distilled water and vacuum freeze-dried. The PBS in the vials was renewed every 2 weeks. The weight of each specimen was measured (WF) and the percentage mass loss was calculated as follow:

1 $${\mathrm{Mass}}\,{\mathrm{loss}}\left(\% \right) = \frac{{{\mathrm{WI}} - {\mathrm{WF}}}}{{\mathrm{WI}}}\times100$$

To assess the products of degradation, an additional sample was kept into PBS for 28 weeks in order to analyze the extracted PBS by means of Raman spectroscopy.

### Biocompatibility tests

Square samples, 10 × 10 mm, were cut from all specimens of electrospun PCL scaffolds, sterilized with 70% ethanol for 6 h put under ultraviolet light for 30 min.

A human Cerebral Microvascular Endothelial Cell Line (Inserm, U1016, Institut Cochin, Paris, France) from 6th Passage was maintained in RPMI 1640 Medium (Roswell Park Memorial Institute) supplemented with 10% fetal bovine serum and 1% penicillin/streptomycin/amphotericin-B (Invitrogen Corp, USA). Cells were maintained in a humidified CO_2_ incubator at 37 °C until confluency and fed with fresh medium every 3 days. Before seeding, cells were detached from the cell-culture flask with trypsin-EDTA and counted using a Neubauer.

MTT (3-(4,5-Dimethylthiazol-2-yl)-2,5-diphenyltetrazolium bromide) cell viability assay method was used to evaluate the biocompatibility of the tissue engineering scaffolding materials. The method is based on the absorbance of the dissolved MTT formazan crystals formed in living cells, which is proportional to the number of viable cells. The electrospun scaffold specimens, after sterilization, were put in empty Polystyrene (PS) well plates. Five hundred microliter of the culture medium was pipetted into each well. For the cell attachment study 2 × 10^4^ cells/well of hCMEC/D3 were seeded and allowed to attach on well plates of PS (control, *n* = 4) and onto scaffolds (treatment, *n* = 4) for 24 and 48 h. After incubation, a calculation of cell viability was followed. In each of all 96 wells, we added 20 μl of the MTT solution (5 mg/ml) in a final volume of 200-μl medium and then incubated for 4 h (to form formazan crystals). Afterwards, the medium was aspirated and 200-μl DMSO was added to each well for the dissolution of formazan crystals. The conversion of MTT to formazan crystals was an indication of the normal state of the cells. Next, a photometric measure at 590 nm was followed using an ELISA photometer. In each well, 200 μl of DMSO was placed and its absorption used as a control. From this point of view, absorption was considered as a percentage of cell viability. Formazan crystals were solubilized by the addition of 100 μl of acidified isopropanol (0.04-N HCl in isopropanol) followed by stirring. Metabolically active cells reacted with tetrazolium salt in the MTT reagent to produce a soluble formazan dye, measured at 620 nm (Micro Plate Reader, MK3, Thermo Lab Systems, USA).

### Fabrication of electrospun tubular scaffolds

**Fig. 1:**
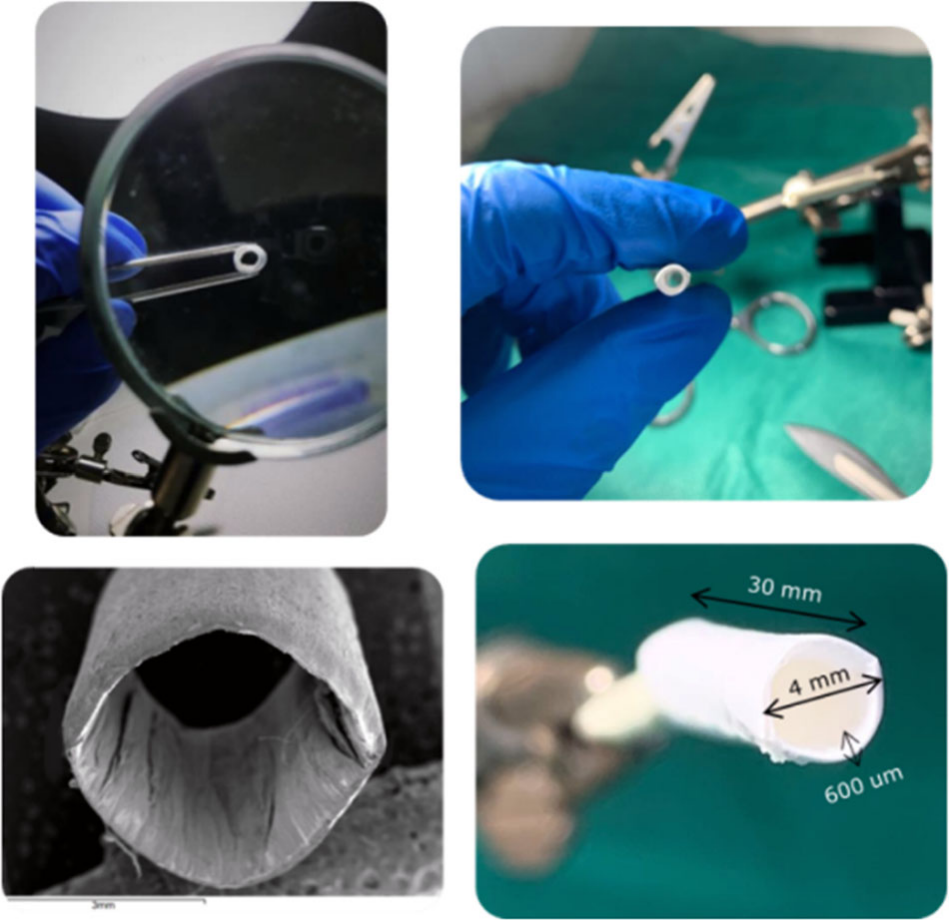
The multilayered polymeric vessel

Rectangular PCL electrospun membrane strips 4 × 30 cm were detached from the peripheral aluminum foil of the cylindrical drum. Fiber alignment was predominately at peripheral orientation of the cylindrical drum, as verified by SEM analysis. The tubular scaffolds were then fabricated through wrapping of the electrospun PCL membrane specimens, cut in a way that fiber alignment being predominately in the longitudinal (axial) direction of the tubular vessel. A multilayer design was chosen in order to mimic the native artery wall structure and match the mechanical properties of natural vessels’ ECM. A surgical cyanoacrylate glue (Dermabond) was utilized in order to fix the ends of the tubular vessel after the wrapping. The final form of the fabricated vessel as well as its dimension can be seen in Fig. 1.

### Mechanical characterization

Assessment of the mechanical properties of the multilayered fibrous polymeric small-caliber scaffold is an important issue, as both, mechanical strength and matching of vascular biomechanics are key parameters (together with biological response) for an increased patency of the scaffold. Uniaxial tests were performed to assess the material anisotropy. In addition, suture retention tests for the safety of anastomosis, cyclic tensile, and stress relaxation testing for the detection of dynamic mechanical stability and viscoelastic performance, as well as tests for the detection of the radial compliance of the tubular scaffolds were performed.

#### Uniaxial tests

The uniaxial mechanical properties of the electrospun fibrous membranes were determined using a bench type miniature tensile tester (MiniMat 2000, Rheometric Scientific Inc.) equipped with a 200-N load cell, at a constant rate of 10 mm/min until rupture [18]. To assess the anisotropy of the scaffolds rectangular specimens 20 × 5 mm were cut along the directional axis of the fibers (the direction of the rotating drum perimeter) and perpendicularly to the fiber axis. Seven (7) samples were tested for each case and the Young’s modulus was assessed. For each specimen, the greatest slope, corresponding to the linear region of the stress–strain curve at a strain range 0–15%, was used to calculate the Young’s modulus.

#### Suture retention strength tests

Suture retention strength tests were performed in 20 × 5-mm rectangular strips of the electrospun membranes (*n* = 7) using the previous testing device. The average thickness of the samples was 500 ± 20 μm. Test protocol was adapted from the methods described within ISO 7198 [26]. A polypropylene surgical suture (4-0, PROPYLEN, medipac^®^) was inserted at 2-mm distance from the end of the sample. The extension rate was set at 100 mm/min, which falls within the range specified by the protocol of the standard. The force required to pull the suture through the sample was recorded with time and the suture retention strength (maximum stress at rupture) was determined.

#### Cyclic tensile tests

Cyclic stability and performance are very important for the elastic expansion and retraction of blood vessels in tissue engineering applications. Although vascular grafts are increasingly developed and exploited, their cyclic mechanical stability, determining their practical applications, is seldom investigated and understood. Ιn order to mimic the effects of repetitive cyclic loading–unloading (similar to that expected in vivo), a set of cyclic loading–unloading mechanical tests was carried out to determine the cyclic mechanical stability of the tubular vessels. An effective way of measuring energy loss is to calculate materials hysteresis. Hysteresis calculations were reported as the total loop area between the loading and unloading curves of the stress–strain diagram divided by the area under the loading curve. The higher the amount of energy loss, the less effective the vascular graft.

Tubular specimens of 20 mm (free length between the grips) and 4-mm internal diameter were fitted in specially designed grips to maintain the tubular form. The tests (*n* = 6) were carried out using a dynamic tensile testing machine (800 LM electromechanical testing device, Test Resources, USA). The specimens were loaded using the sinusoidal loading procedure of the testing machine from 0 to 10% strain at a frequency of 1.25 Hz (i.e., the average heart rate of normal adults at 75 beats per minute). A preload of 0.1 N was used as a “zero state” condition, corresponding to a physiological preloading of the natural vessels. After a 90 cycles preconditioning procedure, the remaining 90–200 cycles of testing were evaluated. The force/elongation of the tubes with time was recorded on-line and the dynamic stress (force/circular ring section area) and strain [(*l* − *l*_0_)/*l*_0_] data and the hysteresis were computed consequently. *L*_0_ was the length of the specimens after the application of 0.1-N preload, considered as the “zero state” of the testing procedure.

#### Stress relaxation tests

Stress relaxation tests were additionally performed after the end of the cyclic testing, for a time which is adequate for a full relaxation of the specimens, in order to evaluate the viscoelastic properties of the tubular vessels. In that frame, the tubular specimens (*n* = 6) were stretched up to 10% strain using a ramp signal procedure (which corresponds to the linear portion of the stress–strain curves obtained from the uniaxial mechanical tests) and held there for 10 min. Stress/time data were computed from force/time on-line data acquisition recordings.

#### Radial compliance measurements

**Fig. 2:**
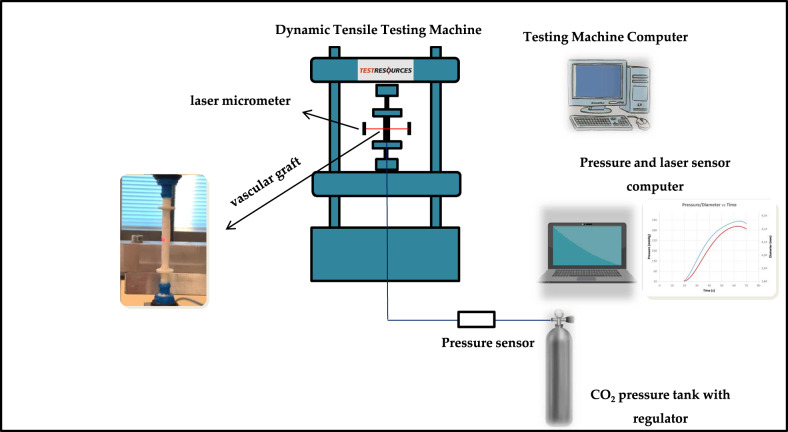
The experimental setup for the radial compliance measurements of the polymeric vessels

Radial compliance measurements were performed following a testing procedure based on recommendations defined in ISO 7198:2016 standard [26], which describes standardized compliance and burst test methods for vascular grafts and scaffolds. For the specific testing procedure, however, the pressure range was limited to slightly above the physiological range. The vascular tubular grafts (*n* = 6) were subjected to a gradually increased internal pressure, and the compliance, i.e., the ability of a prosthesis to elastically expand and contract in the circumferential direction in response to pulsatile pressure, was calculated. Tests were performed in a custom-made setup (Fig. 2), adapted on the same dynamic tensile machine. The internal pressure was applied into the tubular vessel through compressed gas from a CO_2_ chamber and controlled with a pressure regulator. The internal pressure of the tubular vessel was measured at the opposite side via a cannulated T-connector, the third end of which was tampered, using an on-line connected electronic pressure gauge with an operating range of −50–300 mmHg (MLT1199 Transducer/Cable Kit, AD INSTRUMENTS). The diameter change, following the pressure increment, was recorded non-contact using an on-line connected laser micrometer (optoCONTROL 1200, Micro-epsilon). Pressure and diameter data were sent to a programmed data acquisition system, from which the radial compliance was calculated at four different pressure ranges according to the Eq. (2), as described in [26].

2 $$\% \,{\mathrm{Compliance}}/100\,{\mathrm{mmHg}} = \frac{{\frac{{R2 - R1}}{{R1}}}}{{\left( {P2 - P1} \right)}}\times10^4$$

where R2 and R1 are the internal radii at the higher pressure P2 and the lower pressure P1 of each range, respectively.

## Results

### Electrospun scaffold morphology

**Fig. 3:**
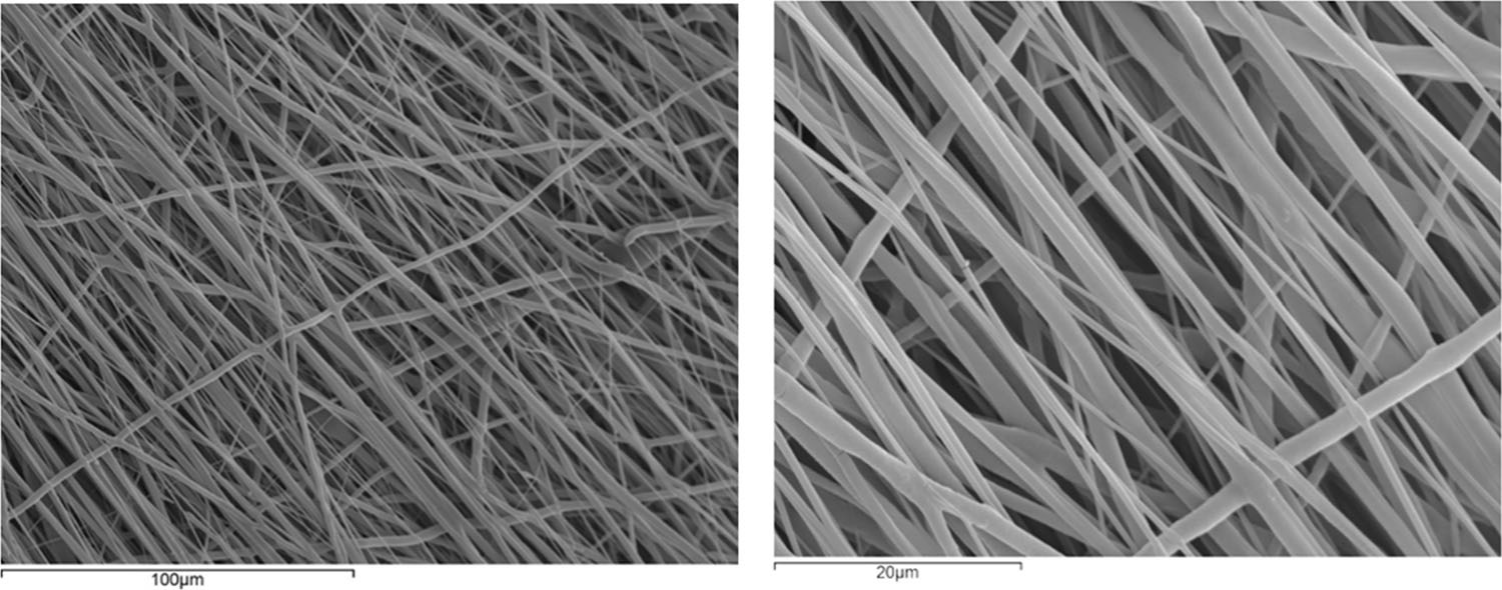
SEM images of the micro-nano-structured electrospun scaffolds

The set of the electrospinning parameters chosen as above (voltage, feed rate, needle to collector distance, rotary collector angular velocity) was found to maintain a stable electrospinning process, leading to a smooth scaffold surface. After a period of 2 h, a membranous scaffold was produced covering the cylindrical surface of the drum collector. The thickness of the scaffolds was measured at different points using a high precision micrometer and found to be average 0.25 ± 0.09 mm (*n* = 20). Figure 3 shows representative SEM images of the electrospun scaffolds. As can be observed, smooth, uniform, defect-free, and round-shaped fibers were produced, and a dominantly parallel alignment of the fibers achieved for the chosen collector velocity. The average fiber diameter was 1.156 ± 0.447 μm (*n* = 50), while the pore size was found to be 17.702 ± 5.369 μm (*n* = 50).

### Wettability of the scaffold

The results of the contact angle measurements showed that the surface of the of the freeze-dried PCL scaffold was very hydrophobic, having an average contact angle of 116° ± 2, *n* = 6. That correlates well with the fact that PCL is known to be a highly hydrophobic material, a property remained unaffected after the electrospinning process.

### In vitro accelerated degradation results

**Fig. 4:**
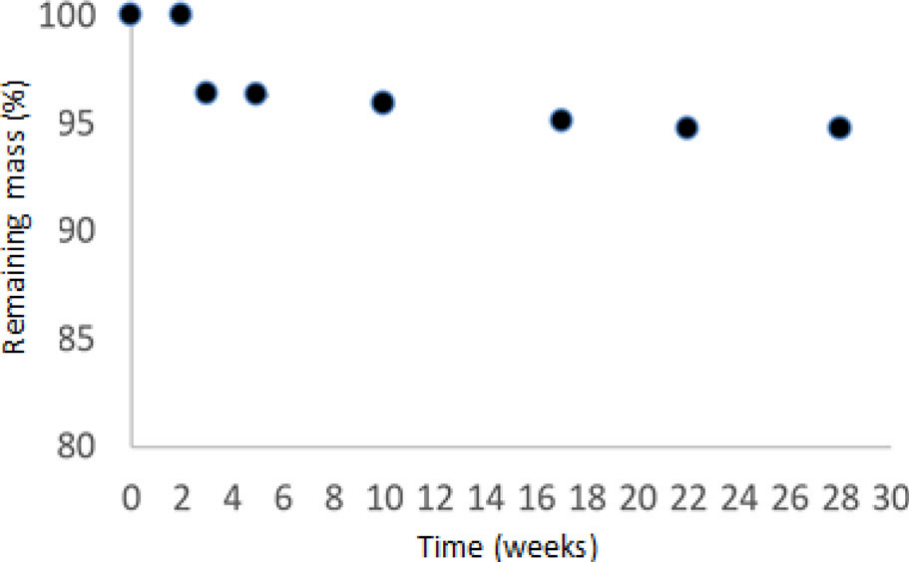
Degradation rate (% remaining mass) of the electrospun PCL membranes

**Fig. 5:**
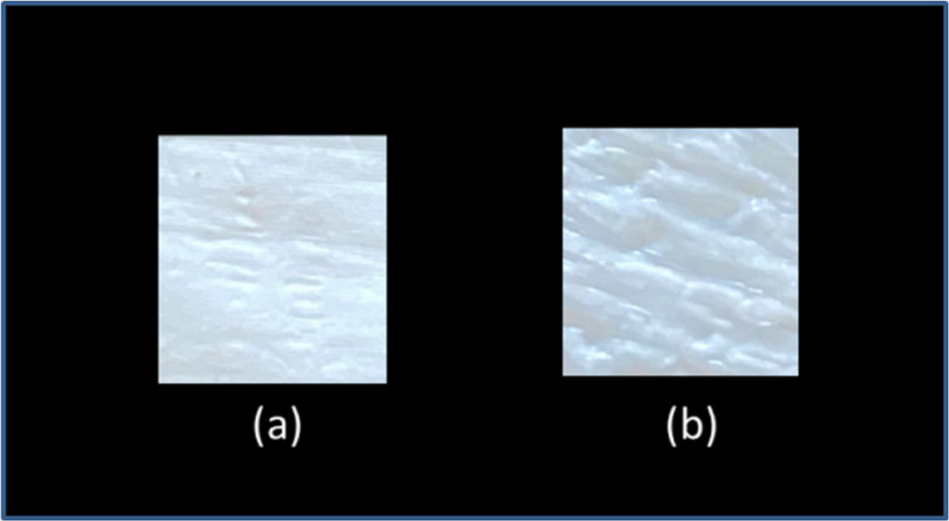
The PCL scaffold at **a** 1st week and **b** 28th week of insertion into the PBS

Results of the in vitro accelerated degradation tests (Fig. 4) showed that there was no significant weight loss till week 3 (~4 %). After this period, the polymeric scaffolds showed a gradual slight increase in degradation till week 17 (~5%), after which, no further weight loss was observed till week 28. Figure 5 shows the surface morphology of the scaffold (a) in the 1st week and (b) after 28 weeks. It can be observed macroscopically that the degradation process occurs at the polymer surface. Use of Raman Spectroscopy on the extracted PBS solution after 28 weeks showed no presence of polymer sub-products, possibly due to very-low concentrations of the PCL products into the solution, lower than the instrument’s sensitivity. The Raman Spectra (not shown here) for the sake of briefness showed not differentiation from that of a typical PBS content.

### Cells viability tests

The results obtained from the MTT assay indicated that electrospun PCL nanofibrous scaffolds were suitable substrates for cell attachment and proliferation. Cytotoxicity was not observed, exhibiting high cell viability (85.64 ± 3.12%) after 2 days.

### Mechanical tests

#### Uniaxial tests

**Fig. 6:**
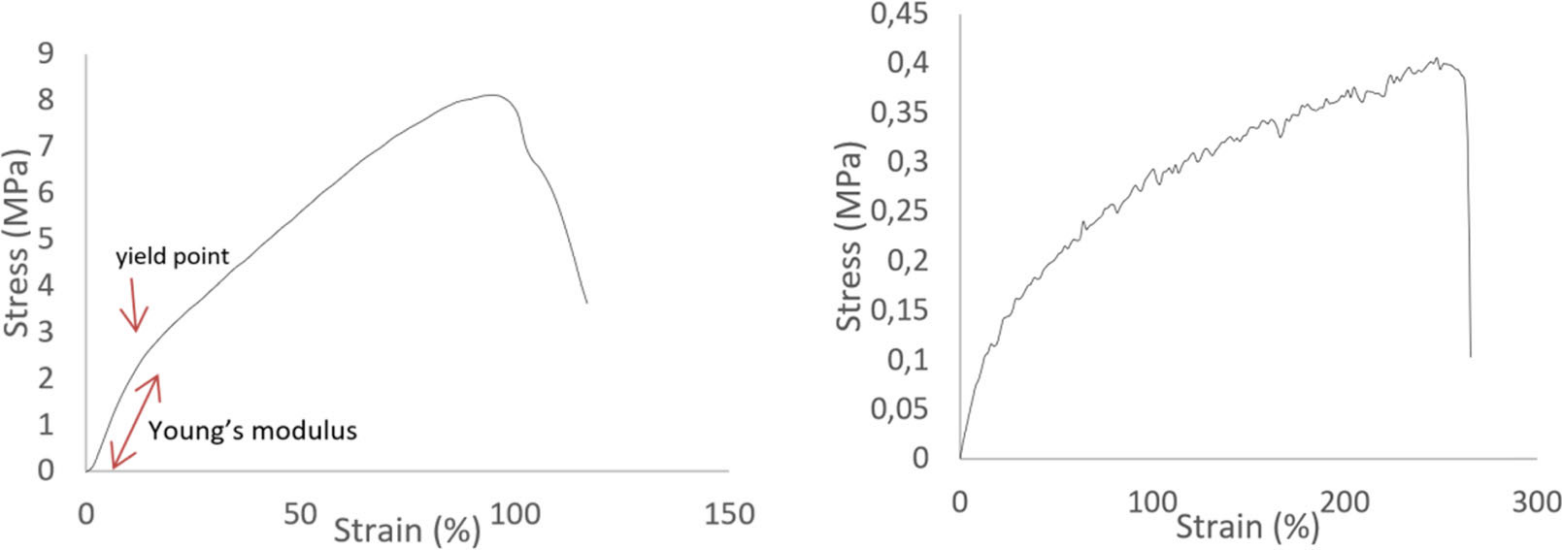
Stress–strain plot of the electrospun PCL membranes **a** parallel and **b** perpendicular to the fiber axis

The average Young’s modulus values (parallel with and perpendicular to the fiber axis) were calculated by analyzing the stress–strain curves. Typical stress–strain curves are depicted in Fig. 6a, b. For both axes, an initial high-slope linear region is observed till the yield point, followed by a second, lower slope linear region, till the rupture of the polymeric scaffold. For the purpose of the study, we considered the first linear region as region of interest, as the strain was into physiological values for small-caliber vessels (<15%). However, a possible explanation for the decrease of the slope in greater strain values can be attributed to the necking of the central region of the specimens and possible micro damages on the fibers, common in polymers at high strain levels. According to the results, a mechanical anisotropy has been attained, as the modulus in the direction parallel with the fiber axis (18 ± 3 Mpa, AVG ± SDV, *n* = 7) was found to be one magnitude higher than that in the perpendicular direction (1 ± 0.3 Mpa AVG ± SDV, *n* = 7). It must be notified that the high modulus corresponds to the axial direction of the tubular scaffold while the lower ones to the circumferential direction.

#### Suture retention strength

**Fig. 7:**
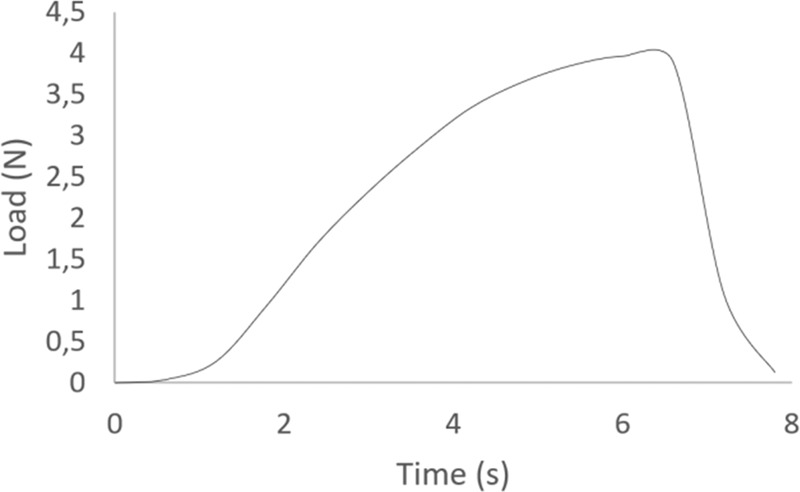
Load-time plot of the sutured PCL electrospun scaffold

A typical load-time plot of the suture retention test is shown in Fig. 7. The suture retention strength of the electrospun PCL scaffold was found to be 4.5 ± 1 N (AVG ± SDV, *n* = 7).

#### Cyclic tensile tests

**Fig. 8:**
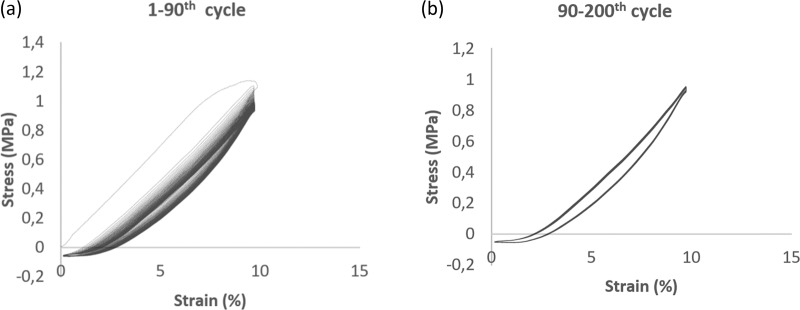
Stress–strain cycle of the tubular polymeric vessel: **a** 1–90th cycle and **b** 90–200th cycle

The cyclic tensile stress–strain curves of the tubular specimens are shown in Fig. 8. In terms of loading and unloading regions, we can see an obvious hysteresis in the first cycle (Fig. 8a). In the following stress–strain cycles, the maximum cycle stress decreases due to the afore-mentioned irreversible hysteresis. Thus, from cycle 2 to cycle 90, there was a gradually increased overlap in the stress–strain curves. This may be attributed to transient rearrangements of the fiber architecture until a stable state, during which a greater part of the loading energy was absorbed. After cycle 90, no further loss of total strain was observed and the cyclic mechanical properties remained stable (Fig. 8b), i.e., the loading and unloading regions are almost identical in each cycle, indicating that the energy stored during each cycle didn’t change. The hysteresis ratio (dissipated energy index) after the 90th cycle was found to be 0.153 ± 0.017 (AVG ± SDV, *n* = 6).

#### Stress relaxation

**Fig. 9:**
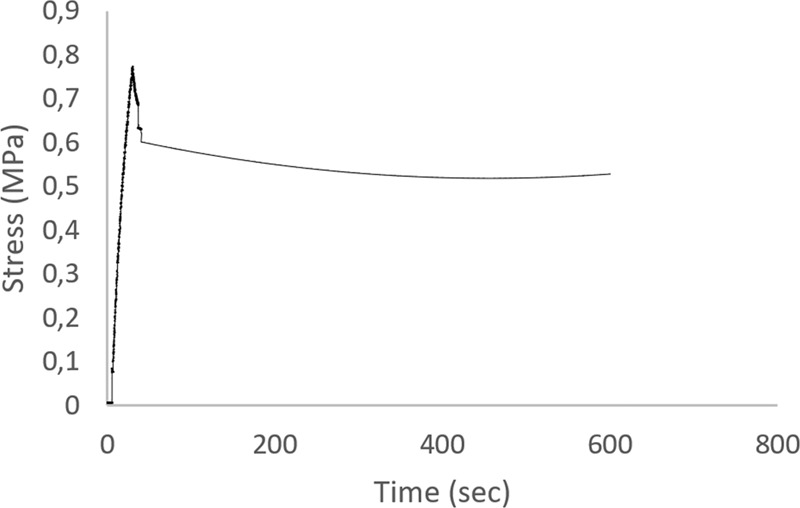
Stress relaxation profile of the polymeric tubular vessel

Figure 9 shows a typical stress relaxation behavior of the tubular vessel at 10% strain. The immediately generated stress gradually decreased with time and reached an equilibrium stress state within a few seconds. The stress drop percentage did not exceed 20% of the initial stress achieved (*n* = 6).

#### Radial compliance measurements

**Fig. 10:**
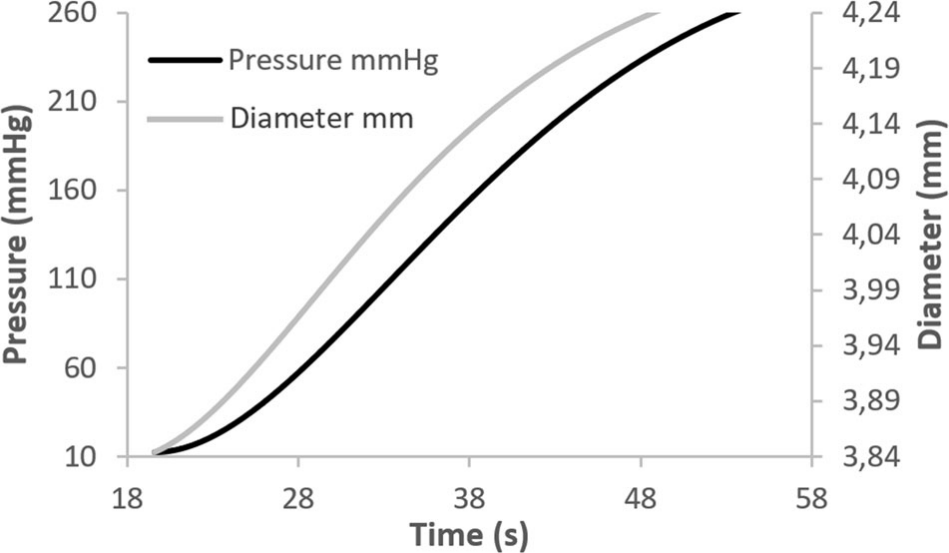
The pressure-diameter profile of the electrospun tubular vessel

Figure 10 shows a typical pressure-diameter versus time plot. From this plot we can observe that diameter increases almost linearly under application of internal pressure into the tubular vessel. The radial compliance measurements for the pressure ranges considered are shown in Table 1. Compliance in the close physiological range 80–120 mmHg was calculated to 5.04 ± 0.82 (AVG ± SDV, *n* = 6).

**Table 1:** Radial compliance values at four different pressure ranges (AVG ± STD, *n* = 6)

Pressure range (mmHg)	Compliance (%)
50–90	7.01 ± 0.94
80–120	5.04 ± 0.82
110–150	4.80 ± 1.06
140–180	4.61 ± 0.96

## Discussion

Currently, there is a continuous research for shelf-ready small-caliber vascular prostheses with satisfactory early and late results, especially in the field of cardio surgery. Aim of the current work was to synthesize small-caliber polymeric (PCL) tissue-engineered vascular scaffolds, in order to mimic the structure and biomechanics of natural vessels. The use of electrospinning process has a great potential to meet the needs for proper synthesis of an appropriate scaffold for medium and small size tissue TEVG. The produced scaffold presented a smooth, uniform, porous, and defect-free fibrous structure, while a dominantly parallel alignment of the fibers was achieved. Fiber alignment and controlled porosity has been found to have a significant effect on cellular behavior in vivo, inducing cell attachment, migration, and differentiation [27]. The achieved fiber diameter of the produced scaffold is comparable to the collagen fibers found in native vessels [28], while its pore size is able to permit cell infiltration through it (e.g., endothelial cells diameter lies between 10 and 20 μm). The gradual loss of weight after the degradation tests of the scaffolds, presented in Fig. 4, can be attributed to PCL hydrolytic degradation due to the presence of hydrolytically labile aliphatic ester linkages [29]; however, the rate of degradation is rather slow (2–3 years). The observed weight loss is in accordance with other studies (e.g., [30]). Our scaffold seems to present a smooth and scheduled degradation in vitro, which is a perquisite for the achievement of a satisfactory functionality and longevity [10, 15]. Finally, cytotoxicity evaluation of the polymeric scaffolds revealed that the materials were nontoxic and did not release substances harmful to living cells, verifying the approval of PCL as a biocompatible polymer.

The differences in structural fiber orientation resulted in analogous mechanical anisotropy of the tubular polymeric scaffolds achieved, as shown in Figs. 3 and 6. The scaffolds were found to be stiffer in axial testing direction than the circumferential one. This is in accordance with vessel mechanical anisotropy encountered in natural arteries and veins [31, 32]. The Young’s modulus found for both directions considered (parallel and perpendicular) match well with those of natural vessels (coronary arteries) as well as autografts used for vascular graft applications, such as the gold standard saphenous vein, and internal mammary artery (IMA) [28, 32–36]. The significant difference observed between the axial and the peripheral direction is very important, as lower peripheral young modulus corresponds to higher peripheral compliance, hence important radial dilatation of the tubular scaffold, a situation not so common in polymeric tubular blood vessel substitutes in current clinical use. Moreover, suture strength of our scaffold, shown in Fig. 7, was found to be superior to that of saphenous vein (around 2.5 N) and IMA (around 2 N) [34, 36, 37], and matched the suture strength of synthetic grafts such as ePTFE [33]. Furthermore, the hysteresis ratio (dissipated energy index), as determined from the hysteresis loop shown in Fig. 8, was found to be comparable with corresponding values (close to 0.18) of native aortic wall [38].

Radial compliance of the polymeric vessels was found to be comparable with compliance data from natural blood vessels (saphenous and umbilical cord veins) [39] and superior to that of synthetic grafts (ePTFE and Dacron) [36, 37]. It is of great interest that compliance decreases as the pressure increases (i.e., vessels become stiffer at higher pressures), a behavior also observed in natural vessels [40]. Radial compliance is critical, because the compliance mismatch between a vascular graft and neighboring arteries at the site of anastomosis is a major cause of graft failure. The main criteria for engineering a long-lasting vessel include, among others, matching compliance and tensile mechanical properties [40]. A prosthetic vessel must be compliant enough to accommodate flow with high pulsatile pressure and tensile enough in order to prevent rupture. In similar studies, mechanical properties of tissue-engineered vessels have been found to be comparable to that of native vessels; however, compliance mismatch was identified as the key factor that limits long-term patency of the graft (e.g., [12, 41–43]). The fact that our polymeric tubular scaffold exhibits biomechanical matching of the radial compliance and tensile properties with natural blood vessels is a very important achievement making it, from a biomechanical point of view, a potential candidate to be used alternatively as vascular implant. However, further in vitro and in vivo tests are required to assess the full tissue regeneration ability of the scaffold.

## Conclusions

During the last two decades, many efforts have been made to develop a functional and mechanically sound tissue TEVG, in order to replace autografts used in coronary bypass surgery. To achieve that, an ideal combination of mechanical, structural, and biological properties is required to make the graft suitable for cardiovascular applications.

In this work, small-caliber polymeric grafts were fabricated through the electrospinning method, with a specific fiber morphology and fiber orientation. The structural architecture and the resulting mechanical properties of the graft were tuned in order to match these of native vessels and arteries. Through in vitro studies and by the use of cell lines, we proved high cells viability on the graft and low cytotoxicity of the polymeric material. The Young’s modulus and suture retention strength of the fabricated grafts were in the range of the ones found in natural vessels. Radial compliance, which is a key determinant for a successful patency of the graft, coincided with the compliance of native vessels (saphenous vein, IMA, etc.) and was found to be superior to the compliance of synthetic grafts (ePTFE, Dacron) in current clinical use.

Although this research illustrates the potential of electrospun vascular grafts for VTE applications, extensive investigation is yet to be conducted, prior to clinical usage. However, the results are very promising toward the use of electrospun vascular grafts for small-caliber vessel replacements. Electrospinning has a great potential to meet the needs for proper design of a polymeric scaffold for small-caliber tissue TEVG. Future work includes combinations of PCL with hydrophilic polymers (e.g., PVA or PGA) to promote host cell access and adhesion in vivo. The results throughout this ongoing research will offer further advancements in the field, necessary before proceeding with extended studies to assess in vitro and in vivo (animal model) cell-scaffold interactions.
